# PSMD12 promotes hepatocellular carcinoma progression by stabilizing CDK1

**DOI:** 10.3389/fimmu.2025.1581398

**Published:** 2025-06-04

**Authors:** Xingyu Peng, Zitao Liu, Chen Luo, Rui Sun, Yuting Zhang, Bowen Li, Yeqing Zou, Jinfeng Zhu, Rongfa Yuan

**Affiliations:** ^1^ Department of General Surgery, The 2^nd^ Affiliated Hospital, Jiangxi Medical College, Nanchang University, Nanchang, Jiangxi, China; ^2^ Jiangxi Medical College, Nanchang University, Nanchang, Jiangxi, China; ^3^ Department of General Surgery, The 1^st^ Affiliated Hospital, Jiangxi Medical College, Nanchang University, Nanchang, Jiangxi, China; ^4^ Pancreas Center, Nanjing BenQ Medical Center, The Affiliated BenQ Hospital of Nanjing Medical University, Nanjing, Jiangsu, China; ^5^ Jiangxi Key Laboratory of Molecular Medicine, The 2^nd^ Affiliated Hospital, Jiangxi Medical College, Nanchang University, Nanchang, Jiangxi, China; ^6^ Hunan Provincial Key Laboratory of the Research and Development of Novel Pharmaceutical Preparations, Changsha Medical University, Changsha, Hunan, China; ^7^ Jiangxi Provincial Clinical Research Center for General Surgery Disease, Nanchang, Jiangxi, China

**Keywords:** PSMD12, hepatocellular carcinoma, Cdk1, deubiquitination, cell cycle

## Abstract

Proteasome 26S subunit non-ATPase 12 (PSMD12), a critical subunit of the proteasome system, is essential for maintaining protein homeostasis. However, its role in hepatocellular carcinoma (HCC) remains underexplored. Bioinformatics analysis, immunohistochemistry, Western blotting, and qRT-PCR confirmed the upregulation of PSMD12 in HCC tissues compared to normal liver tissues, with this overexpression correlating with poor patient prognosis. Functional assays revealed that PSMD12 knockdown suppressed HCC cell proliferation and migration, inducing G2/M phase cell cycle arrest. In contrast, PSMD12 overexpression promoted these malignant behaviors. Mechanistically, PSMD12 interacts with cyclin-dependent kinase 1 (CDK1), preventing its degradation through deubiquitination, thereby accelerating HCC progression by enhancing cell cycle progression. These findings underscore PSMD12’s role in HCC and highlight its potential as both a prognostic biomarker and therapeutic target, providing new insights into the molecular mechanisms driving HCC progression.

## 1 Introduction

According to the 2022 Global Cancer Statistics, liver cancer ranks as the third leading cause of cancer-related deaths worldwide (1). Approximately 90% of primary liver cancers are classified as hepatocellular carcinoma (HCC). The clinical management of HCC is particularly challenging due to its subtle onset, aggressive invasiveness, and poor prognosis (2). Therefore, elucidating the complex molecular mechanisms driving HCC development and identifying novel therapeutic targets are essential for improving patient outcomes and advancing treatment strategies.

Deubiquitination, a post-translational modification primarily mainly by deubiquitinating enzymes (DUBs), plays a critical role in various cellular processes (3). DUBs prevent the degradation of target proteins via the ubiquitin-proteasome system (UPS), or by removing ubiquitin from substrates (4). Proteasome 26S subunit non-ATPase 12 (PSMD12), also known as Rpn5, is an ATP-independent component of the 19S regulatory subunit, facilitating deubiquitination of trapped substrates (5, 6). Recent studies have demonstrated elevated PSMD12 expression in various tumor types, correlating with poor clinical outcomes (7–9). However, the precise role of PSMD12 in HCC and its underlying mechanisms remain unclear. Dysregulated cell cycle regulation is a significant factor in tumor progression (10). Cyclin-dependent kinase 1 (CDK1), a member of the cell cycle-regulated protein kinase family, plays a pivotal role in the G1 and G2/M phases (11, 12). The dysregulation of CDK1 has been closely associated with tumor progression, with elevated CDK1 levels observed in breast, stomach, colorectal, and liver cancers (13–16).

This study identified abundant PSMD12 expression in HCC cells, which enhanced cell proliferation and migration. Flow cytometry analysis revealed that silencing PSMD12 led to G2/M phase cell cycle arrest. Further investigation revealed that PSMD12 interacts with CDK1, stabilizing it through deubiquitination, thereby promoting HCC progression.

## 2 Materials and methods

### 2.1 Bioinformatics analysis

Expression data for expression levels of PSMD12 in both non-cancerous and cancerous tissues derived from HCC specimens were retrieved downloaded and analyzed using several publicly available databases, including The Cancer Genome Atlas Program (TCGA, https://portal.gdc.cancer.gov/), Gene Expression Omnibus (GEO, GSE1212148, GSE14520; https://www.ncbi.nlm.nih.gov/geo/), and the International Cancer Genomics Consortium (ICGC, https://dcc.icgc.org/). Kaplan–Meier survival analysis for PSMD12 in HCC was performed using the Kaplan–Meier Plotter (http://kmplot.com/analysis/). Differential expression analysis of PSMD12 in HCC was conducted using the UALCAN online tool. SangerBox (http://vip.sangerbox.com/home.html) was utilized to visualize differential expression and prognostic data for PSMD12 in a pan-cancer context. The list of abbreviations and their definitions are presented in **Supplementary Table 1**.

### 2.2 Patients and tissue specimens

HCC tissue samples were collected from 32 patients who underwent resection at the Department of General Surgery, Second Affiliated Hospital of Nanchang University, China. None of the patients had undergone chemotherapy before surgery. All participants provided written informed consent, and the study adhered to the ethical standards outlined in the Declaration of Helsinki. Approval was obtained from the Ethics Committee of the Second Affiliated Hospital of Nanchang University.

### 2.3 Cell culture and transfection

Human HCC cell lines HepG2 (SCSP-510) and Huh-7 (SCSP-526) were sourced from the National Collection of Authenticated Cell Cultures, Chinese Academy of Sciences. Additional human HCC cell lines HCCLM3 (CL-0042) and SMCC7721 (CL-0216) were obtained from Procell (Wuhan, China), and the MHCC97H (ZB001) cell line was acquired from Bei Zhi Creatures (Shanghai, China). The human immortalized liver immortalized cell line THLE-2 (KL388h) was provided by Kang Lang Biology (Shanghai, China). All cell lines were authenticated by short tandem repeat profiling and were confirmed free from mycoplasma contamination. Cells were cultured in a complete culture medium containing 10% fetal bovine serum, maintained at 37°C with 5% CO2. ShRNA and overexpression plasmids, along with the corresponding lentiviral vectors for transfection, were supplied by Hanheng Biotechnology (Shanghai, China) and Focus Bioscience (Nanchang, China). Transient transfections were performed using Lipofectamine™ 3000 (Invitrogen, Carlsbad, CA, USA) according to the manufacturer’s instructions. Stable cell lines were established by selecting lentivirus-transfected cells with puromycin.

### 2.4 RNA extraction and quantitative real-time PCR

Following previous research (17), RNA was extracted from tissue samples or cultured cells using RNAiso Plus (Takara, Japan), then the RNA was reverse transcribed to cDNA, and amplified using PCR. Primer sequences are provided in **Supplementary Table 2**.

### 2.5 Western blot, immunofluorescence, hematoxylin-eosin, and immunohistochemistry staining

Western blot, IF, H&E staining, and IHC were performed following established protocols (18–20). The following primary antibodies were utilized in this study: anti-mouse PSMD12 (Proteintech), anti-rabbit CDK1 (Proteintech), anti-rabbit PLK1 (Abclonl), anti-rabbit Phospho-PLK1-T210 (p-PLK1, Abclonl), anti-rabbit AKT (Abclonl), anti-rabbit Phospho-AKT-S473 (p-AKT, Abclonl), anti-rabbit Cyclin D1 (Proteintech), anti-mouse PCNA (Santa Cruz Biotechnology), anti-mouse GAPDH (Proteintech), anti-rabbit Ki-67 (Abcam), anti-rabbit PSMD12 (Santa Cruz Biotechnology), anti-mouse Ki-67 (Abcam), anti-rabbit PSMD12 (Santa Cruz Biotechnology), anti-mouse CDK1 (Santa Cruz Biotechnology) and anti-mouse Ubiquitin (Proteintech).

### 2.6 Co-immunoprecipitation and ubiquitination test

For the Co-IP assay, cell lysates were incubated overnight at 4°C with specific primary antibodies, followed by incubation with protein A/G sepharose beads (Santa Cruz Biotechnology). After three washes with lysis buffer, coprecipitated proteins were eluted and analyzed by Western blot using the indicated antibodies. For mass spectrometry, samples underwent processing via liquid chromatography-tandem mass spectrometry (LC-MS/MS). To minimize potential interference from the light chain of the immunoprecipitating antibody, a goat anti-rabbit secondary antibody (Abmart, 1:1000) was used for CDK1 Western blot analysis. A goat anti-mouse secondary antibody (Abmart, 1:1000) was used for PSMD12 Western blot to avoid interference from the heavy chain.

For the ubiquitination assay, cells were transfected and treated with the proteasome inhibitor MG132 (15 μmol/L, MedChemExpress) and the protein synthesis inhibitor cycloheximide (CHX, 50 μg/mL, MedChemExpress) for 6 hours. Following treatment, cells were lysed using the protocol employed for the Co-IP assay, and immunoprecipitation with anti-CDK1 or anti-PSMD12 antibodies was conducted, followed by Western blot analysis.

### 2.7 Cell proliferation assays

In the colony formation assay, stable transfection cell groups were seeded at a density of 500 cells per well in 6-well plates. The medium was replaced every three days. After 14 days, colonies were fixed with 4% paraformaldehyde, stained with crystal violet, and imaged. Colony counts were quantified using ImageJ software.

For the CCK-8 assay, cells were seeded into 96-well plates at a density of 3,000 cells per well and incubated at various time points (0 h, 24 h, 48 h, and 72 h). Cells were then incubated with a 1.5-hour treatment of CCK-8 reagent (UElandy) diluted with a complete medium (1:9). Absorbance was measured at 450 nm.

For the assay using the YF^®^594 Click-iT EdU Staining Kit (UElandy), cells were treated with 100 µL of 1x Apollo reaction cocktail for one hour after washing three times with PBS. Cells were then stained with Hoechst 33342 to assess DNA content. Images were captured using an Olympus fluorescence microscope (Olympus, Tokyo) and analyzed using ImageJ software.

### 2.8 Cell migration assays

Cells were seeded in 6-well plates at a density of 4×10^5^ cells per well. Once reaching approximately 90% confluence, a sterile 200-μL pipette tip was used to create a uniform scratch across the cell monolayer. Detached cells and debris were gently washed twice with PBS, and cultures were maintained in a serum-free medium. Images of the scratch area were captured at 0, 24, and 48 hours using an inverted microscope.

For the transwell migration assay, treated HCC cells (2×10^4^ cells) were seeded into the upper chamber of Transwell inserts (8 μm pore size, Corning) with serum-free medium. The lower chamber was filled with a complete medium containing 10% FBS as a chemoattractant. After 24 hours of incubation, non-migrated cells on the upper surface of the membrane were gently removed with a cotton swab. Migrated cells on the lower surface were fixed with 4% paraformaldehyde, stained with crystal violet, and imaged under a light microscope. Cell counts were performed using ImageJ software.

### 2.9 Flow cytometry assay

For cell cycle analysis, exponentially growing HCC cells were digested with trypsin without EDTA, centrifuged at 200×g for 5 minutes, washed twice with ice-cold PBS, and fixed in 70% ice-cold ethanol overnight. After removing the ethanol, the cells were resuspended in propidium iodide staining solution from the Cell Cycle Kit (UElandy) and incubated in the dark. Cell cycle distribution was analyzed using flow cytometry.

### 2.10 Subcutaneous xenograft experiments

Four-week-old female BALB/c nude mice were purchased from Hangzhou Zhiyuan Laboratory Animal Technology Co., Ltd. MHCC97H cells (5×10^6^) stably transfected with shNC, shPSMD12#1, shNC+CDK1, or shPSMD12#1+CDK1 were resuspended in 200 μL of PBS and subcutaneously injected into the dorsal region of the nude mice. Tumor size was measured every 4 days using vernier calipers. Tumor volume was calculated using the formula: V = 0.5×length×(width)^2^, where length is the longest diameter and width is the shortest diameter. At the end of the observation period, mice were euthanized by inhalation of carbon dioxide gas. Tumors were dissected, weighed, and fixed in 4% paraformaldehyde for IHC analysis. All animal experiments were approved by the Animal Ethics Committee of Nanchang Royo Biotech Co., Ltd (Approval No. RYE2022091402).

### 2.11 Prediction of protein-protein interaction between PSMD12 and CDK1 based on AlphaFold3

The amino acid sequences of CDK1 (UniProt ID: P06493) and PSMD12 (UniProt ID: Q96P21) were retrieved from the UniProt database (https://www.uniprot.org/). AlphaFold3 was then employed to predict their complex structure using default parameters. The resulting protein-protein complex was subsequently imported into PyMOL for three-dimensional visualization. Using PyMOL-generated structural models, the interaction interface was systematically analyzed to identify critical interfacial residues and characterize the specific interaction patterns within this region.

### 2.12 Statistical analysis

Data are presented as mean ± standard error of the mean (SEM) using GraphPad Prism 8.0 (GraphPad Software, USA). Each experiment was independently replicated three times. For comparisons between two groups, a paired or unpaired Student’s t-test was applied. For comparisons among multiple groups, one-way analysis of variance (ANOVA) was used, followed by Tukey’s multiple comparison test for pairwise comparisons. The comparison of PSMD12 expression between pan-cancer tissues and corresponding normal tissues was analyzed using unpaired Wilcoxon Rank Sum and Signed Rank Tests. Prognostic differences between high and low PSMD12 expression groups were analyzed using the Log-rank test. Uni- and multivariate Cox regression analyses were employed to assess prognostic factors. Spearman’s correlation analysis was performed to evaluate the correlation between two factors. Statistical significance was defined as a p-value of less than 0.05.

## 3 Results

### 3.1 PSMD12 was overexpressed in HCC and associated with poor clinical prognosis in patients

**Figure 1:**
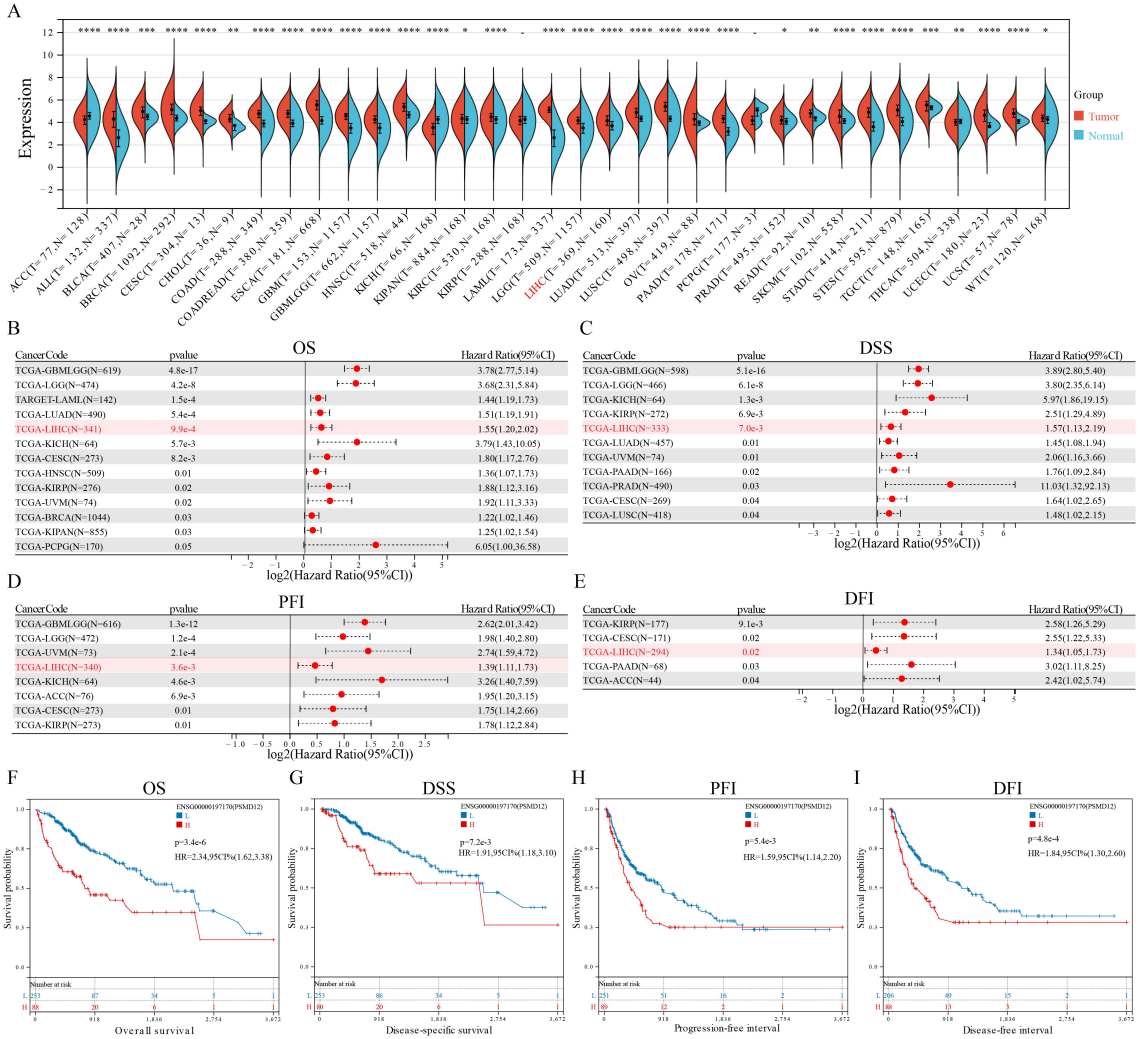
PSMD12 expression and its prognostic significance across pan-cancer. **(A)** PSMD12 expression across pan-cancer was obtained from the TCGA+GTEx database and analyzed using the Wilcoxon rank-sum and signed-rank tests. **(B-E)** Correlation of PSMD12 expression with overall survival (OS), disease-specific survival (DSS), disease-free interval (DFI), and progression-free interval (PFI) in pan-cancer patients. Prognostic significance was evaluated through the log-rank test, with only statistically significant data presented. **(F-I)** Comparison of OS, DSS, DFI, and PFI between high and low PSMD12 expression groups (log-rank test). “L” and “H” denote low and high expression groups. *p < 0.05, **p < 0.01, ***p < 0.001, ****p < 0.0001.

The integration of the TCGA and GTEx databases revealed overexpression of PSMD12 was overexpressed in various cancers, including ALL, BLCA, BRCA, CESC, CHOL, COAD, COADREAD, ESCA, GBM, GBMLGG, HNSC, KIPAN, KIRC, LAML, LGG, LIHC, LUAD, LUSC, OV, PAAD, PRAD, READ, SKCM, STAD, STES, TGCT, UCEC and WT (**Figure 1A**). In contrast, lower expression levels of PSMD12 were observed in ACC, KICH, and THCA compared to corresponding normal tissues (**Figure 1A**). The correlation between PSMD12 expression and patient prognosis in various cancers was evaluated using the log-rank test. The results demonstrated that elevated PSMD12 expression was associated with reduced overall survival (OS) in patients with GBMLGG, LGG, LAML, BRCA, CESC, LUAD, KIRP, KIPAN, HNSC, LIHC, UVM, and PCPG (**Figure 1B**). High PSMD12 expression correlated with shorter disease-specific survival (DSS) in patients with GBMLGG, LGG, KICH, KIRP, LIHC, LUAD, UVM, PAAD, PRAD, CESC, and LUSC (**Figure 1C**). Additionally, high PSMD12 expression was associated with a shorter progression-free interval (PFI) in patients with GBMLGG, LGG, UVM, LIHC, KICH, ACC, CESC, and KIRP (**Figure 1D**). Similarly, high PSMD12 expression correlated with shorter disease-free intervals (DFI) in patients with KIRP, CESC, LIHC, PAAD, and ACC (**Figure 1E**). Kaplan-Meier survival curves were plotted to illustrate the differences in OS, DSS, DFI, and PFI between patients with high and low PSMD12 expression levels in the TCGA database, demonstrating that elevated PSMD12 expression correlates with poorer prognoses (**Figures 1F–I**).

**Figure 2:**
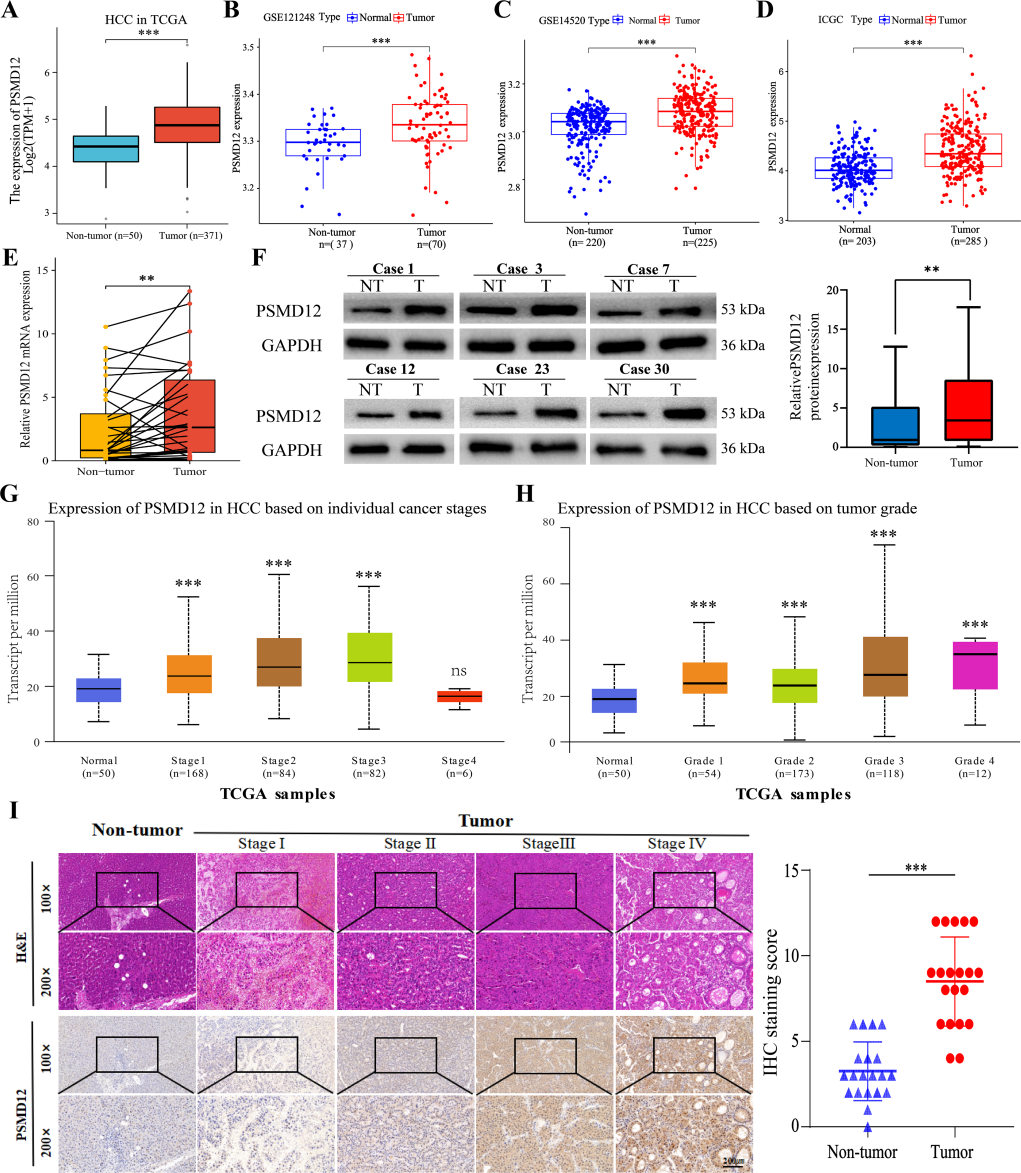
PSMD12 expression in HCC with experimental validation. **(A-D)** mRNA expression of PSMD12 in non-tumor and HCC samples was compared across TCGA, GSE121248, GSE14520, and ICGC datasets (unpaired Student’s t-test). **(E)** mRNA levels of PSMD12 in HCC and non-tumor tissues were assessed by qRT-PCR (n = 32, paired Student’s t-test). **(F)** Western blot analysis was conducted to assess PSMD12 protein expression in both HCC and corresponding non-tumor tissues (n = 32), with GAPDH as the loading control. **(G-H)** PSMD12 expression in the TCGA-HCC cohort was mapped across various stages **(G)** and grades **(H)** of HCC using the UALCAN tool. **(I)** Representative H&E and IHC staining of PSMD12 in HCC tissues at various stages, and in non-tumor samples, at 100×magnification with insets at 200×magnification. Scale bar: 200 mm. Statistical analysis of IHC staining scores for PSMD12 in HCC and paired non-tumor tissues (n = 20). Paired two-tailed Student's t-tests were applied to matched tumor and non-tumor tissues, with **p < 0.01, ***p < 0.001; ns, not significant.

To further investigate PSMD12 expression in HCC, datasets from TCGA, GEO, and ICGC were analyzed. Results showed a significant increase in PSMD12 expression in tumor tissues compared to non-tumor tissues in TCGA (**Figure 2A**), GSE121248 (**Figure 2B**), GSE14520 (**Figure 2C**), and ICGC (**Figure 2D**). Additionally, qRT-PCR and Western blot analysis of HCC patient samples from our hospital confirmed that PSMD12 expression was significantly elevated at both the mRNA and protein levels (**Figures 2E, F**). The UALCAN database revealed that PSMD12 expression increased with the stage and grade of HCC (**Figures 2G, H**), and these findings were further validated by IHC analysis of HCC patient specimens from our hospital (**Figure 2I**). Both univariate and multivariate Cox regression analyses performed on the TCGA cohort identified PSMD12 as an independent prognostic factor for poor outcomes in HCC (p = 0.047, **Table 1**). These results collectively indicate that PSMD12 is highly expressed in HCC and is closely associated with unfavorable patient outcomes.

**Table 1:** Univariate and multivariate regression analysis of PSMD12 expression and clinical parameters in TCGA-HCC patients.

Characteristics	Total(N)	Univariate Analysis		Multivariate Analysis	
		Hazard Ratio (95% CI)	P Value	Hazard Ratio (95% CI)	P Value
Age	370				
<= 60	177	Reference			
> 60	193	1.248 (0.880 - 1.768)	0.214		
Gender	370				
Female	121	Reference			
Male	249	0.816 (0.573 - 1.163)	0.260		
Pathologic T stage	367				
T1&T2	274	Reference		Reference	
T3&T4	93	2.540 (1.785 - 3.613)	**< 0.001**	1.857 (0.252 - 13.697)	0.544
Pathologic N stage	256				
N0	252	Reference			
N1	4	2.004 (0.491 - 8.181)	0.333		
Pathologic M stage	270				
M0	266	Reference		Reference	
M1	4	4.032 (1.267 - 12.831)	**0.018**	2.716 (0.803 - 9.189)	0.108
Pathologic stage	346				
Stage I&Stage II	256	Reference		Reference	
Stage III&Stage IV	90	2.449 (1.689 - 3.549)	**< 0.001**	1.414 (0.192 - 10.406)	0.734
PSMD12	370				
Low	185	Reference		Reference	
High	185	1.557 (1.098 - 2.208)	**0.013**	1.576 (1.006 - 2.469)	**0.047**

Bold represents differences that are statistically different.

### 3.2 PSMD12 promotes HCC cell proliferation and migration

**Figure 3:**
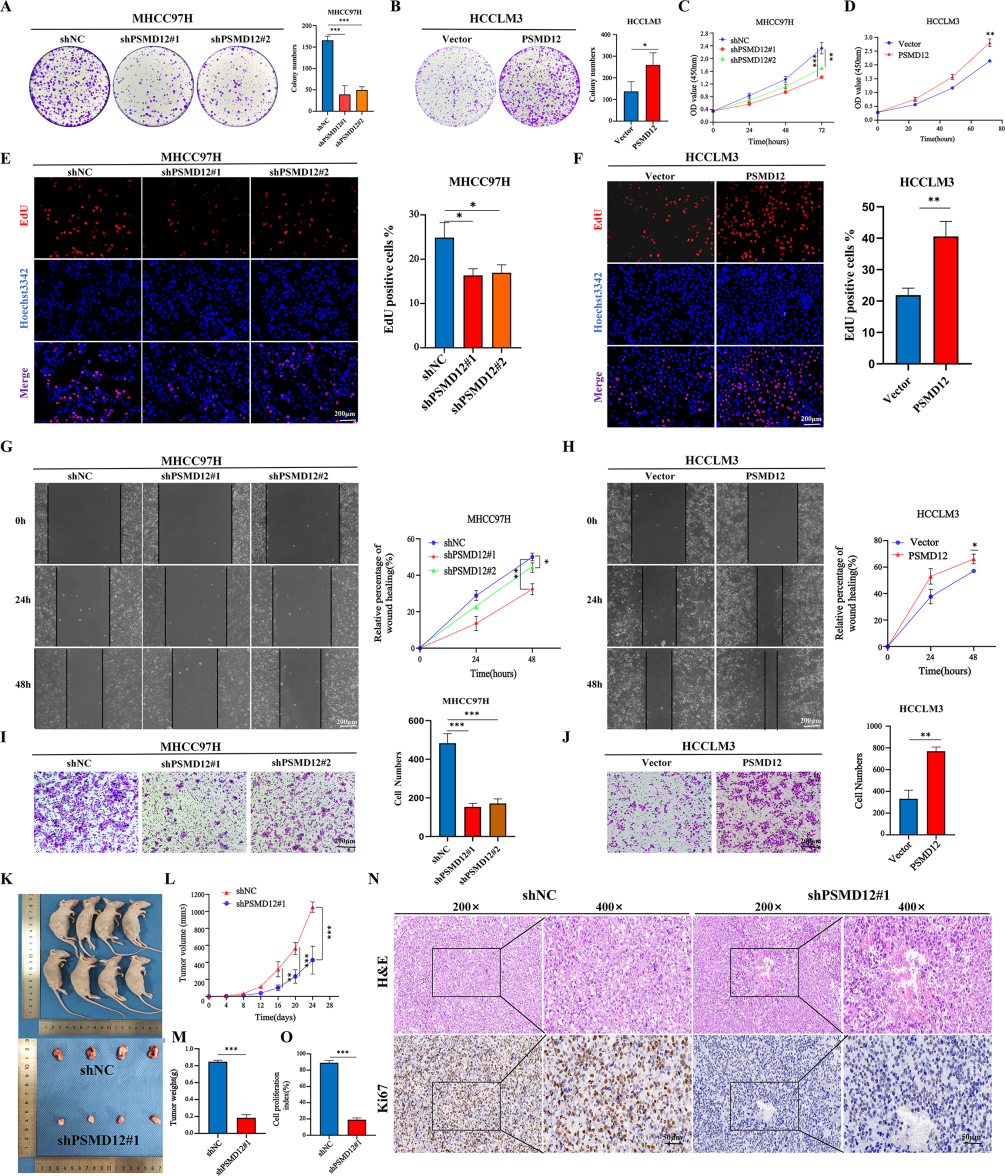
Effects of PSMD12 on HCC cell growth and migration. **(A, B)** Representative images and quantitative analysis of colony formation assays in hepatocellular carcinoma cells after PSMD12 knockdown or overexpression. **(C-D)** CCK-8 experiment was performed to measure the cell viability. **(E, F)** EdU assay demonstrating the proliferative capacity of cells with varying PSMD12 expression levels (scale bar: 200 μm). **(G, H)** Representative images of wound-healing assays in hepatocellular carcinoma cells following knockdown or overexpression of PSMD12 (scale bar: 200 μm). **(I, J)** Representative images of transwell assays in HCC cells following PSMD12 knockdown or overexpression (scale bar: 200 μm). Data are presented as mean ± standard deviation from three independent experiments. For two groups, Student’s t-tests were applied; for more than two groups, one-way ANOVA with Tukey’s multiple comparisons test was used (*p < 0.05, **p < 0.01, ***p < 0.001; ns, not significant). **(K-M)** MHCC97H (shNC or shPSMD12#1) cells were subcutaneously injected into BALB/c nude mice. Tumor volumes were measured at specified time points, and at the experiment’s conclusion, tumors were excised, photographed, and weighed. Tumor volume (V) was calculated as V = 0.52 × length × width2. Tumor volume and weight were analyzed using one-way ANOVA followed by Tukey’s test. Tumor volumes are presented as mean ± SD, n = 4, **p < 0.01, ***p < 0.001. **(N, O)** Representative H&E staining showcases tumor tissues extracted from shPSMD12 and control nude mice. Tumor tissues were subjected to Ki67 staining, and the cell proliferation index was determined by quantifying Ki67-positive nuclei (n = 4, magnification: 200 ×, inset magnification: 400 ×; ***p < 0.001. Scale bar: 50μm).

Expression levels of PSMD12 were analyzed in various cell lines using qRT-PCR and Western blot. The results indicated higher mRNA and protein levels of PSMD12 in HCC cell lines compared to the immortalized human hepatocyte line THLE-2 (**Supplementary Figures S1A, B**). Among the HCC cell lines, PSMD12 expression was highest in MHCC97H and lowest in HCCLM3 (**Supplementary Figures S1A, B**). To further investigate the functional role of PSMD12, knockdown, and overexpression plasmids were further screened for subsequent experiments (**Supplementary Figures S1C–F**). Colony formation assays demonstrated that PSMD12 knockdown significantly inhibited the proliferative capacity of MHCC97H cells, while PSMD12 overexpression significantly enhanced the proliferation of HCCLM3 cells (**Figures 3A, B**). Consistent results were observed in CCK-8 and EdU assays (**Figures 3C-F**). Wound-healing assays showed that PSMD12 regulates cell migration, as knockdown in MHCC97H cells reduced migration (**Figure 3G**), while overexpression in HCCLM3 cells enhanced migration (**Figure 3H**). These results were further corroborated by transwell migration assays (**Figures 3I, J**). To evaluate the *in vivo* impact of PSMD12 modulation on tumorigenesis, subcutaneous xenograft experiments were conducted using female BALB/c nude mice. Four weeks post-cell inoculation, tumors from PSMD12-stable knockdown mice exhibited significantly smaller size and weight compared to the control group (**Figures 3K–M**). Ki-67 staining confirmed a reduction in cell proliferation within the PSMD12-knockdown tumors (**Figures 3N, O**).

### 3.3 PSMD12 promotes HCC cell growth by facilitating the cell cycle

**Figure 4:**
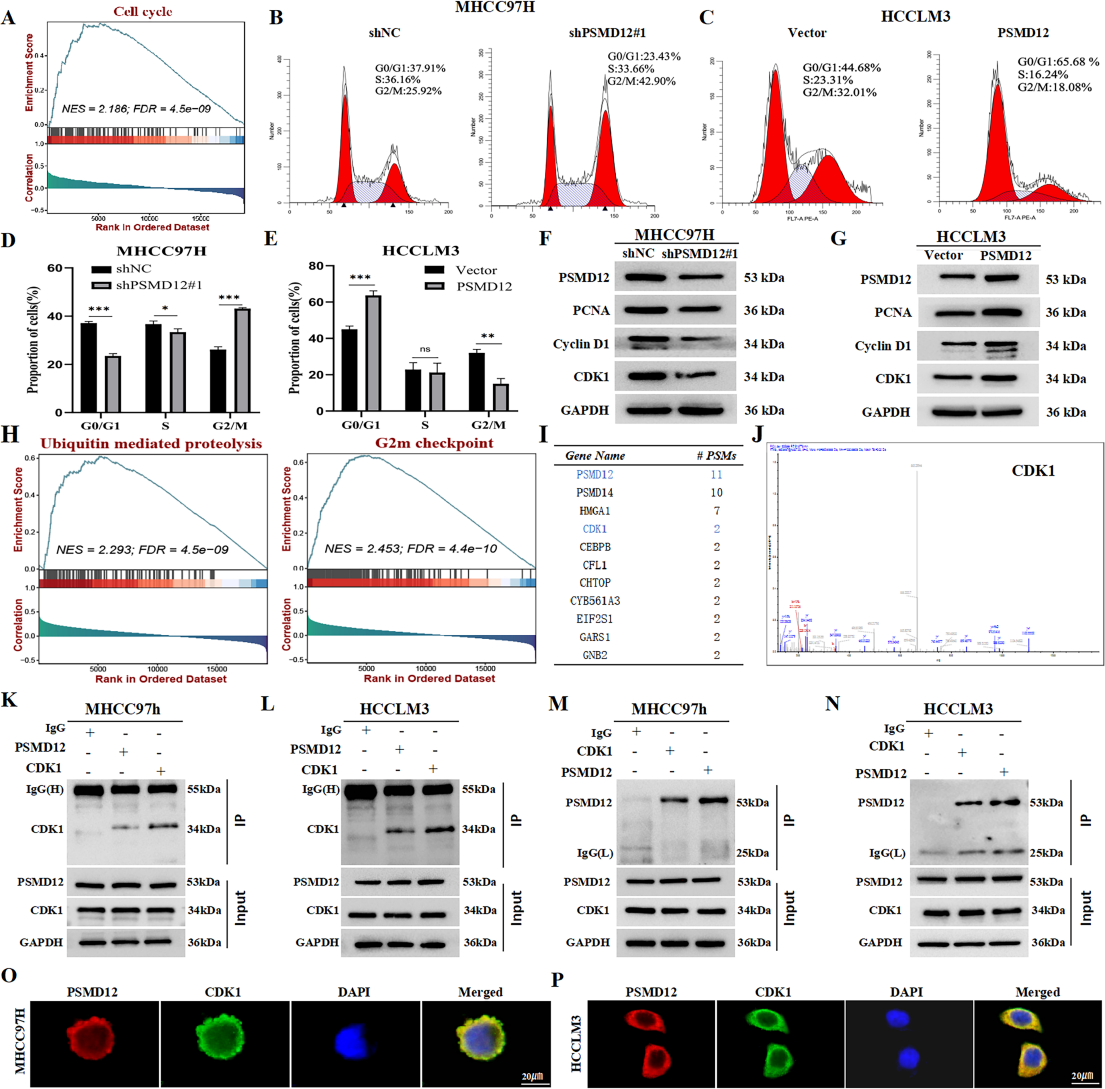
PSMD12 promotes HCC cell cycle progression and interacts with CDK1. **(A)** GSEA analysis of the TCGA-HCC cohort highlighting cell cycle signaling pathways. **(B-E)** Cell cycle distribution in HCC cells following PSMD12 knockdown or overexpression, presented as peak plots and quantitative analysis of cell distribution across G0/G1, S, and G2/M phases. **(F, G)** Western blot analysis of PSMD12, PCNA, Cyclin D1, CDK1, and GAPDH protein expression in PSMD12-silenced MHCC97H cells **(F)** and PSMD12-overexpressing HCCLM3 cells **(G)**, with GAPDH as the control. **(H)** GSEA analysis of the TCGA-HCC cohort revealing the Ubiquitin-mediated proteolysis and G2/M checkpoint signaling pathways. **(I, J)** Mass spectrometry detected co-precipitated PSMD12 and CDK1 proteins. **(K-N)** Co-immunoprecipitation (Co-IP) assays in MHCC97H and HCCLM3 cells confirmed the interaction between PSMD12 and CDK1. **(O, P)** Co-localization of PSMD12 (red) and CDK1 (green) in HCC cells, followed by DAPI nuclear counterstaining (blue). Scale bar: 20 μm. *p < 0.05, **p < 0.01, ***p < 0.001; ns, not significant.

To elucidate the mechanisms by which PSMD12 influences HCC cell progression, gene set enrichment analysis (GSEA) was conducted using the TCGA HCC dataset. The analysis revealed a significant association between PSMD12 and cell cycle regulation (**Figure 4A**). Flow cytometry analysis of cell cycle progression showed that PSMD12 knockdown in MHCC97H cells increased the proportion of cells in the G2/M phase while decreasing those in the G0/G1 and S phases, compared to the shNC group. Conversely, PSMD12 overexpression in HCCLM3 cells increased the G0/G1 phase population and reduced the G2/M phase population (**Figures 4B–E**). Western blot analysis of cell cycle-related proteins demonstrated that PSMD12 knockdown led to reduced expression of PCNA, Cyclin D1, and CDK1 (**Figure 4F**), while PSMD12 overexpression upregulated these proteins (**Figure 4G**). Further GSEA indicated a strong correlation between PSMD12 and ubiquitin-mediated proteolysis pathways, as well as the G2/M checkpoint (**Figure 4H**). Immunoprecipitation of MHCC97H cell lysates with an anti-PSMD12 antibody followed by mass spectrometry identified CDK1, a key G2/M checkpoint kinase, as a potential interacting partner (**Figures 4I, J**). Co-immunoprecipitation confirmed direct interactions between PSMD12 and CDK1 in HCC cells (**Figures 4K-N**). Additionally, confocal microscopy-based immunofluorescence staining validated the co-localization of PSMD12 and CDK1 in HCC cells (**Figures 4O, P**).

### 3.4 PSMD12 promotes HCC progression through CDK1

**Figure 5:**
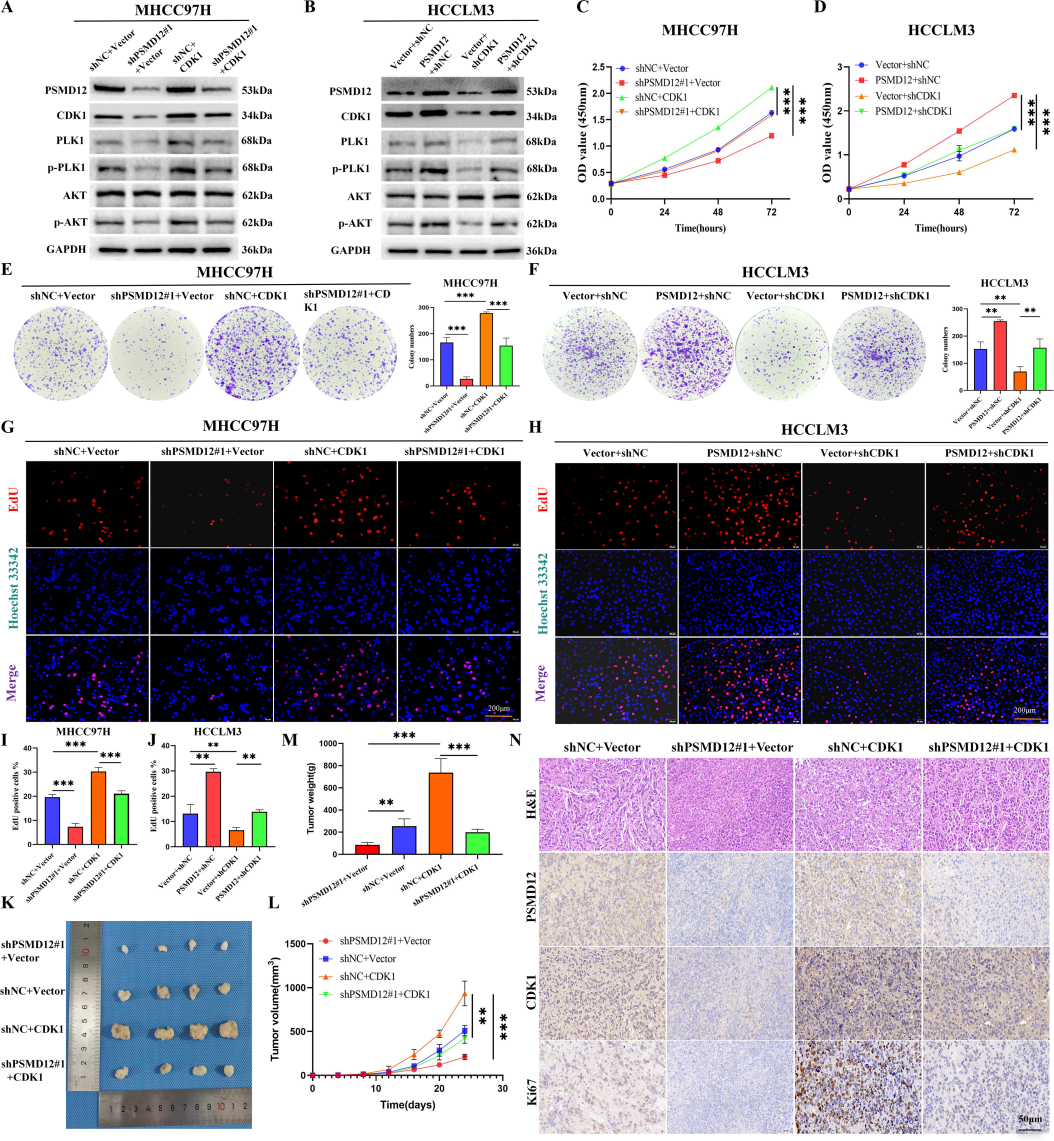
PSMD12 regulates HCC proliferation through CDK1. **(A)** Western blot analysis of PSMD12, CDK1, PLK1, p-PLK1, AKT, p-AKT, and GAPDH expression in MHCC97H cells transfected with shNC, Vector, shPSMD12#1, or CDK1. **(B)** Western blot analysis showing protein expression of PSMD12, CDK1, PLK1, p-PLK1, AKT, p-AKT, and GAPDH in HCCLM3 cells transfected with Vector, PSMD12, or shCDK1. **(C)** CCK-8 assays demonstrated that restoration of CDK1 expression counteracted the growth-inhibitory effect of PSMD12 knockdown in MHCC97H cells. **(D)** Knockdown of CDK1 expression inhibited the pro-proliferative effect induced by PSMD12 overexpression in HCCLM3 cells, as assessed by CCK-8. **(E, F)** Representative images and quantification of colony formation assays in hepatocellular carcinoma cells following PSMD12 or CDK1 knockdown or overexpression. **(G, H)** EdU assays were performed to assess the proliferative capacity of cells (scale bar: 200 μm). **(I, J)** Quantification of EdU assays in hepatocellular carcinoma cells following PSMD12 or CDK1 knockdown or overexpression. Data are presented as mean ± standard deviation from three independent experiments and analyzed using one-way ANOVA followed by Tukey’s multiple comparisons test. **p < 0.01, ***p <0.001. **(K)** Representative tumor morphology in BALB/c nude mice. **(L, M)** Statistical analysis of tumor volume **(L)** and tumor weight **(M)** across different groups. Tumor volume (V) was calculated as V = 0.52 × length × width2, and data were analyzed by one-way ANOVA followed by Tukey’s multiple comparisons test. Tumor volumes are presented as mean ± SD, n = 4, **p < 0.01, ***p < 0.001. **(N)** Representative H&E and IHC staining of PSMD12, CDK1, and Ki67 in tumor tissues from different nude mouse groups. Scale bar: 50 μm.

**Figure 6:**
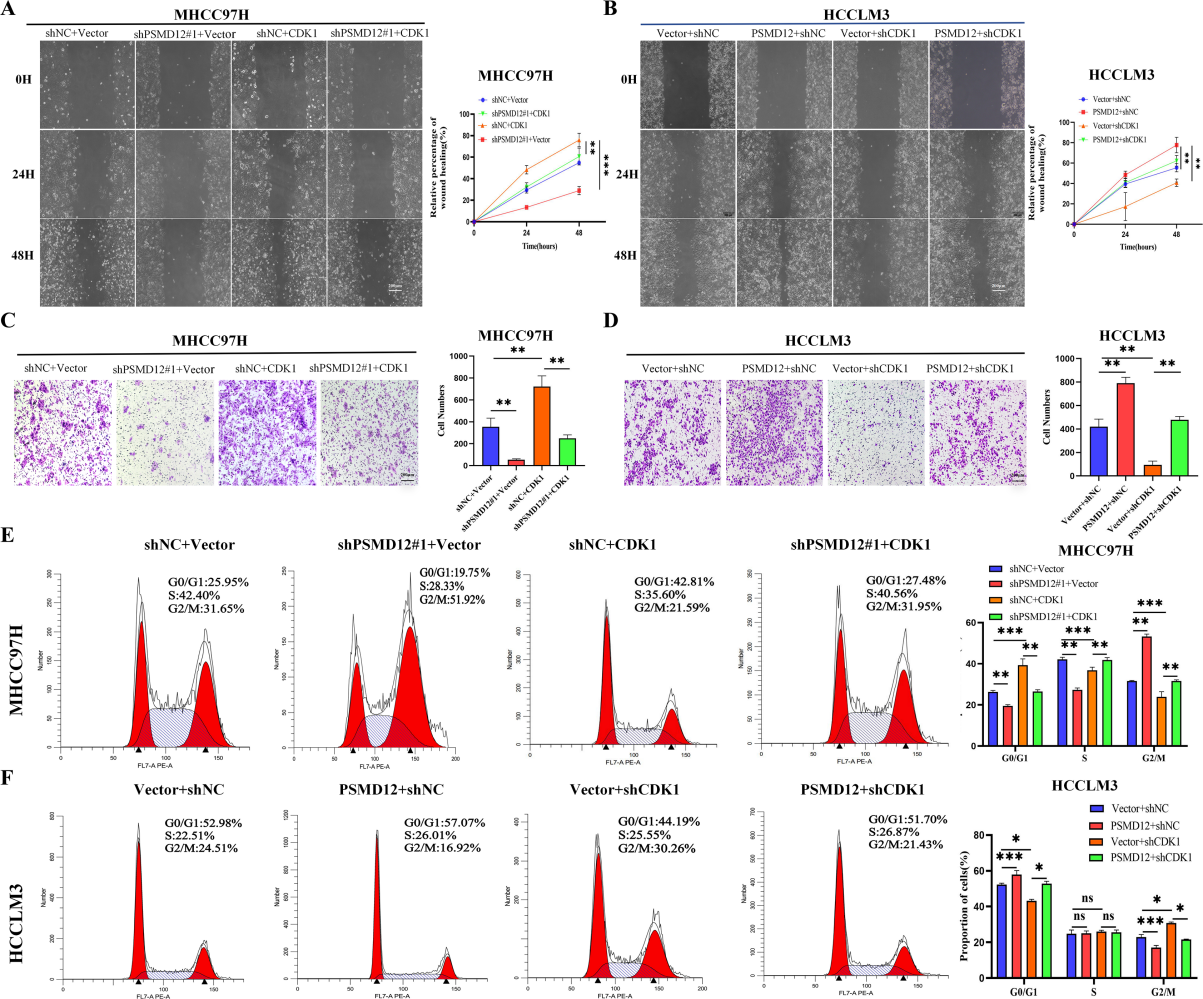
PSMD12 regulates the HCC cell cycle and migration through CDK1. **(A, B)** Representative images and quantification of wound-healing assays in various groups. **(C, D)** Transwell assays were employed to assess cell invasive capacity. **(E, F)** Representative images and quantification of cell cycle distribution in different groups. Data are presented as mean ± SD from three independent experiments. Statistical significance was determined using one-way ANOVA followed by Tukey’s multiple comparisons test. *p < 0.05, **p < 0.01, ***p < 0.001; ns, not significant.

Further GSEA showed that genes related to PSMD12 were significantly enriched in the PI3K/AKT/mTOR signaling pathway(**Supplementary Figures S2A, B**). Integrating these findings, we hypothesized that PSMD12 might exert its function through CDK1, acting on both the G2/M checkpoint regulatory network and the PI3K/AKT/mTOR signaling cascade, thus influencing the progression of HCC. Rescue experiments validated this hypothesis. In MHCC97H cells (**Figure 5A**), the knockdown of PSMD12 significantly inhibited the expression and phosphorylation level of PLK1. Meanwhile, the phosphorylation of AKT was also suppressed. However, when CDK1 was overexpressed against the background of PSMD12 knockdown, it effectively reversed the downward trend of the above-mentioned indicators. In contrast, in HCCLM3 cells (**Figure 5B**), overexpression of PSMD12 significantly promoted the expression, phosphorylation of PLK1, and phosphorylation of AKT. Conversely, the silencing of CDK1 counteracted the upregulatory effects of PSMD12 overexpression on these indicators. CCK-8 assays revealed that elevated CDK1 expression counteracted the reduced growth of MHCC97H cells following PSMD12 knockdown (**Figure 5C**), while CDK1 inhibition reversed the enhanced proliferation of HCCLM3 cells induced by PSMD12 overexpression (**Figure 5D**). Similar findings were obtained from colony formation (**Figures 5E, F**) and EdU staining assays (**Figures 5G, J**). *In vivo*, experiments showed that CDK1 restoration alleviated the tumor growth suppression caused by PSMD12 knockdown (**Figures 5K–M**), with IHC staining of tumor tissues from nude mice further corroborating these results (**Figure 5N**). Wound healing assays revealed that excess CDK1 expression counteracted the migration impairment of MHCC97H cells caused by PSMD12 reduction (**Figure 6A**), while CDK1 inhibition in HCCLM3 cells abrogated the enhanced migration resulting from PSMD12 overexpression (**Figure 6B**), consistent with transwell migration assays (**Figures 6C, D**). Flow cytometry analysis indicated that CDK1 upregulation in MHCC97H cells with PSMD12 knockdown reduced G2/M phase arrest and increased the proportion of cells in G0/G1 and S phases (**Figure 6E**). Conversely, CDK1 downregulation in HCCLM3 cells diminished the increase in G0/G1 and S phase cells caused by PSMD12 overexpression, leading to a higher number of cells arrested in the G2/M phase (**Figure 6F**). The above results reveal that in HCC cells, PSMD12 can dynamically regulate the PLK1 (G2/M) and AKT signaling pathways by regulating the expression of CDK1. This regulatory mechanism plays a central role in key biological behaviors of HCC cells, such as cell cycle progression, proliferation, and migration.

### 3.5 PSMD12 maintains CDK1 protein stability via the ubiquitin-proteasome pathway

**Figure 7:**
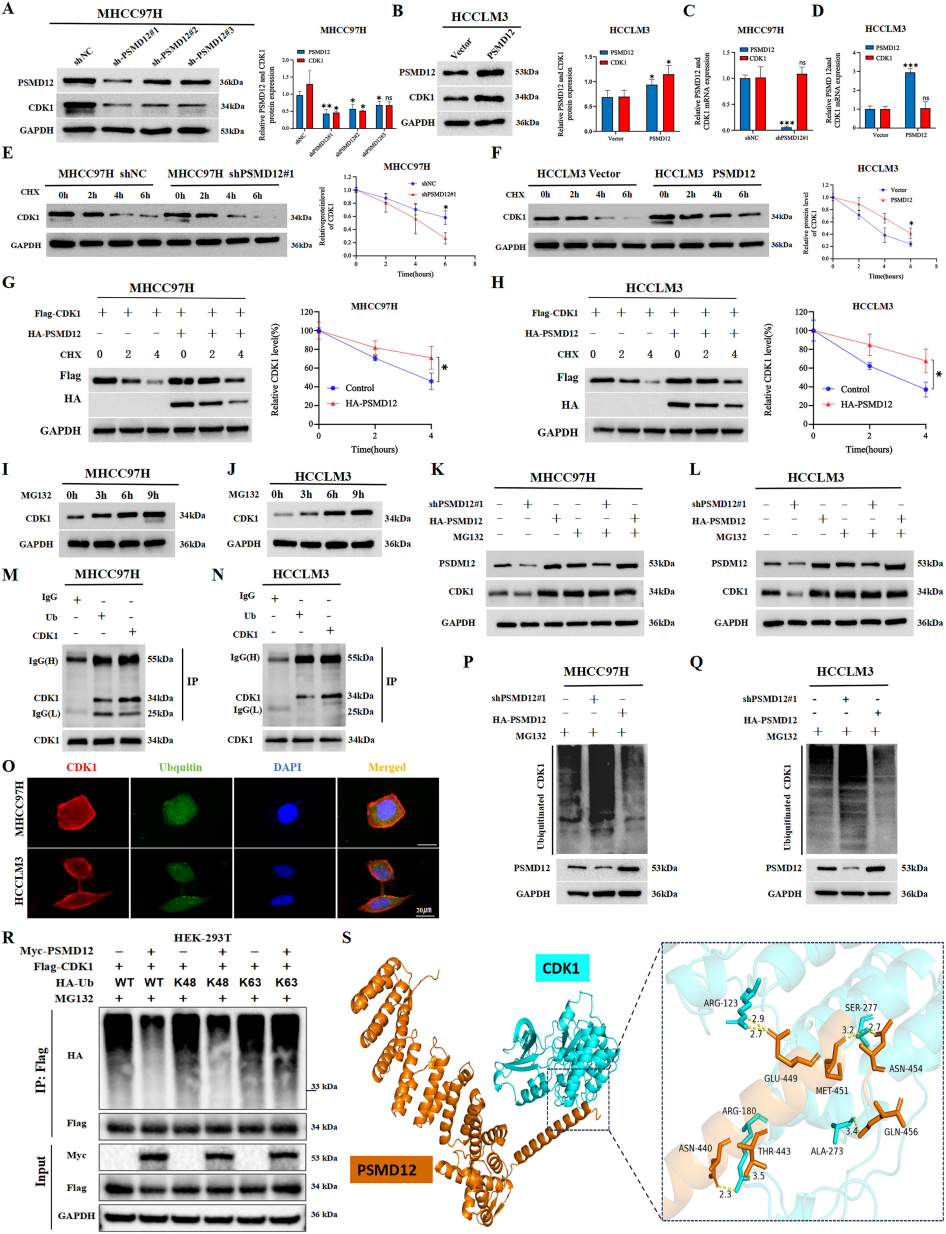
PSMD12 stabilizes CDK1 protein levels by reducing ubiquitin-mediated degradation of CDK1 in HCC cells. **(A, B)** Western blot analysis of PSMD12 and CDK1 protein levels in HCC cells following PSMD12 knockdown or overexpression, with GAPDH as a loading control. **(C, D)** qRT-PCR assessment of PSMD12 and CDK1 mRNA levels in HCC cells after PSMD12 knockdown or overexpression, with GAPDH as a control. **(E, F)** MHCC97H and HCCLM3 cells were treated with cycloheximide (CHX, 50 μg/mL), and Western blot analysis was performed to detect CDK1 protein levels at different time points. **(G, H)** MHCC97H and HCCLM3 cells were treated with CHX (50 μg/mL) for specified durations, with or without the addition of the PSMD12 overexpression plasmid, followed by Western blot analysis to assess CDK1 protein levels. **(I, J)** HCC cells were treated with 15 μmol/L MG132 and collected at 0/3/6/9 hours, followed by Western blot to analyze protein expression levels. **(K, L)** MG132 (15 μM) treatment of MHCC97H and HCCLM3 cells altered PSMD12 expression, with PSMD12 and CDK1 protein levels assessed by Western blot analysis. **(M, N)** Co-immunoprecipitation (Co-IP) assays in MHCC97H and HCCLM3 cells confirmed the interaction between ubiquitin (Ub) and CDK1. **(O)** Colocalization studies in HCC cells using an anti-CDK1 antibody (1:100, red) and anti-ubiquitin antibody (1:100, green), followed by DAPI nuclear counterstaining (blue). Scale bar: 20 mm. **(P, Q)** MG132 (15 μM) was added to MHCC97H and HCCLM3 cells, which were simultaneously transfected with shPSMD12#1 or HA-PSMD12 plasmids. Co-IP assays were performed to detect ubiquitin binding to CDK1. **(R)** After 48 hours of transfection of Myc-PSMD12, Flag-CDK1, and HA-Ub WT/K48/K63, cells were lysed, and then immunoprecipitation was performed with Flag antibody, and immunoblot analysis was performed with HA antibody. **(S)** The docking conformation and three-dimensional structure of PSMD12 and CDK1.PSMD12 and CDK1 are shown in orange and cyan respectively. Statistical significance was determined using Student's t-tests and one-way ANOVA followed by Tukey's multiple comparisons test. *p < 0.05, **p < 0.01, ***p < 0.001; ns, not significant.

To dissect the molecular mechanism of PSMD12-CDK1 interaction, we first confirmed that PSMD12 modulates CDK1 protein expression without affecting its mRNA levels in HCC cells (**Figures 7A–D**), indicating a post-translational regulatory role. Treatment with the protein synthesis inhibitor CHX revealed that PSMD12 silencing shortened the half-life of endogenous CDK1 (**Figure 7E**), while PSMD12 overexpression prolonged it (**Figure 7F**). Consistent results were observed with exogenous protein constructs (**Figures 7G, H**), suggesting PSMD12 regulates CDK1 stability. Using the proteasome inhibitor MG132, we demonstrated that CDK1 degradation occurs via the ubiquitin-proteasome pathway, as MG132 treatment led to CDK1 accumulation (**Figures 7I, J**). Importantly, in PSMD12-depleted or -overexpressing cells, MG132 abrogated the effect of PSMD12 on CDK1 levels (**Figures 7K, L**), confirming dependency on the proteasomal pathway. Co-IP assays showed that endogenous CDK1 is ubiquitinated in HCC cells (**Figures 7M, N**), with immunofluorescence revealing significant co-localization between CDK1 and ubiquitin (**Figure 7O**). Co-IP results demonstrated that PSMD12 knockdown increased CDK1 ubiquitination, whereas PSMD12 overexpression decreased it (**Figures 7P, Q**), indicating that PSMD12 stabilizes CDK1 by inhibiting its ubiquitination. Mechanistically, PSMD12 specifically reduced K48-linked polyubiquitination of CDK1, a key marker for proteasomal degradation (21) (**Figure 7R**). Molecular docking analysis further visualized the direct interaction interface between PSMD12 and CDK1 (**Figure 7S**). Key hydrogen bonds were identified: ARG123 of CDK1 formed bonds with GLU449 of PSMD12 (2.9 Å and 2.7 Å), and SER277 of CDK1 interacted with ASN454 of PSMD12 (3.2 Å and 2.7 Å). Spatial proximity between ARG180 of CDK1 and ASN440/THR443 of PSMD12, as well as ALA273 of CDK1 and MET451/GLN456 of PSMD12, suggested potential hydrophobic interactions or van der Waals forces, stabilizing the complex structure. Collectively, these hydrogen bonds, electrostatic forces, and hydrophobic contacts stabilize the PSMD12-CDK1 complex structure.

### 3.6 Positive correlation between PSMD12 and CDK1 expression in HCC tissues

**Figure 8:**
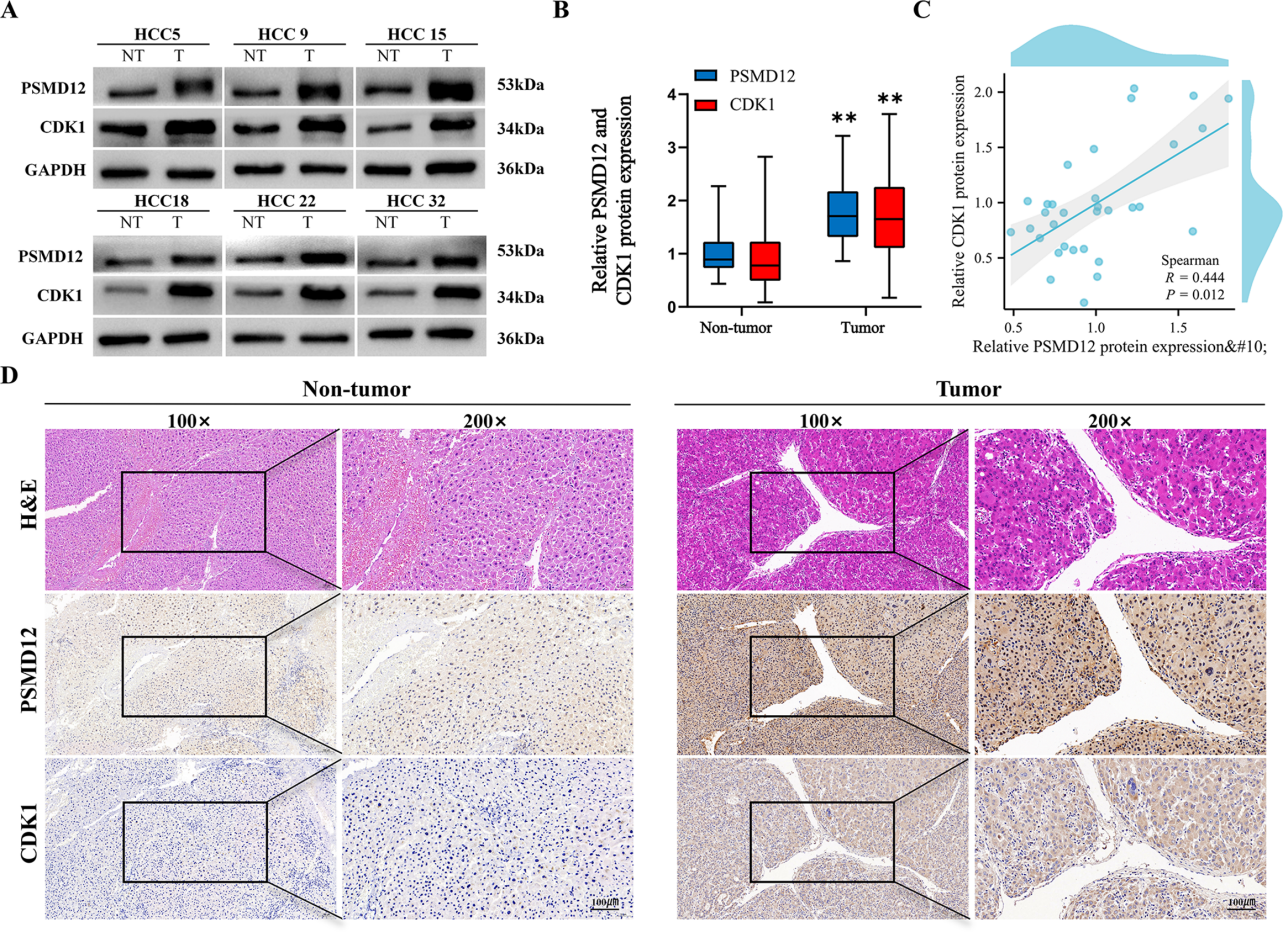
Validation of PSMD12 and CDK1 protein expression. **(A, B)** Western blot analysis was used to assess PSMD12 and CDK1 protein levels in HCC tissues and corresponding non-tumor tissues **(A)** and to quantify the results **(B)**, with GAPDH as a loading control. Statistical significance was determined using a paired two-tailed Student’s t-test, **p < 0.01. **(C)** Scatter plot illustrating the correlation between PSMD12 and CDK1 protein levels in HCC samples. Spearman’s correlation analysis was performed (n = 32, r = 0.444, p = 0.012). **(D)** Representative immunohistochemical (IHC) staining of PSMD12 and CDK1 in HCC tissues (magnification: 100 ×, inset). IHC staining of PSMD12 and CDK1 in corresponding non-tumor tissues (magnification: 100 ×, inset at 200 × magnification, scale bar: 100 μm).

Clinical correlation analysis revealed that both PSMD12 and CDK1 were upregulated in HCC tissues compared to normal liver tissues (**Figures 8A, B**). Statistical analysis showed a significant positive correlation between their expression levels (R = 0.444, p = 0.012; **Figure 8C**), validated by IHC staining (**Figure 8D**). These findings link the PSMD12-CDK1 regulatory axis to human hepatocarcinogenesis.

## 4 Discussion

Protein ubiquitination, a critical post-translational modification, plays a pivotal role in cellular processes. Recent studies have increasingly highlighted the role of ubiquitination and deubiquitination in regulating metabolic changes in cancer cells (22). Furthermore, accumulating evidence has demonstrated the significant involvement of ubiquitination and deubiquitination in the onset and prognosis of HCC (23, 24). The PSMD family is closely associated with tumor development through its regulation of ubiquitination and deubiquitination processes. For instance, the depletion of PSMD1 leads to the ubiquitination of cellular proteins, causing cell cycle arrest and eventually triggering cell death in cancer cells (25). Luo et al. reported that PSMD7 promoted pancreatic cancer progression by stabilizing SOX2 expression through deubiquitination (17), and Lv et al. found that PSMD14 facilitated HCC growth and metastasis by regulating deubiquitination (26). However, the role of PSMD12 in HCC remains underexplored, with its molecular mechanisms yet to be fully elucidated. Our study indicates that PSMD12 is highly expressed in HCC tissues and cells, correlating with poor patient prognosis. Additionally, our findings suggest that PSMD12 contributes to deubiquitination and regulates cell cycle progression in HCC cells.

The serine/threonine protein kinases, particularly the CDK family, are frequently dysregulated in cancer, disrupting the cell cycle and promoting tumor growth. As a central member of the CDK family, CDK1 has been increasingly recognized for its involvement in HCC progression, immune cell infiltration, and patient prognosis (27, 28). Mounting evidence indicates that activated CDK1 promotes both the transcription and phosphorylation of PLK1, with phosphorylated PLK1 further driving G2/M transition and cell cycle progression through multiple mechanisms (29–31). Notably, recent studies have revealed that activated PLK1 in tumors can directly phosphorylate AKT, thereby potentiating tumor malignancy (32, 33). Previous studies have demonstrated the roles of both PSMD12 and CDK1 in promoting HCC progression. However, the potential interaction between PSMD12 and CDK1 remains unclear. We found that both PSMD12 and CDK1 are highly expressed and positively correlated in HCC cells. Furthermore, we discovered the CDK1-PLK1-AKT signaling axis regulated by PSMD12, which underscores its crucial role in the mitotic progression and oncogenic signaling in HCC. However, the specific mechanism through which PSMD12 affects CDK1 remains undefined. Previous reports have shown that CDK1 regulation occurs via deubiquitination. For example, YOD1 stabilizes CDK1 through deubiquitination, promoting tumorigenesis in triple-negative breast cancer (34). Ci et al. found that OTUD4 facilitates glioblastoma progression by deubiquitinating CDK1 and activating the MAPK signaling pathway (35), while Liu et al. demonstrated that USP14 regulates breast cancer cell cycle progression by removing ubiquitination from CDK1 (21). This study found that PSMD12 stabilizes CDK1 by specifically removing the K48-linked polyubiquitination of CDK1.

Despite the novel insights into the PSMD12-CDK1 regulatory axis in HCC, this study has several limitations. First, although we demonstrated that PSMD12 stabilizes CDK1 by reducing K48-linked polyubiquitination, the specific lysine residues on CDK1 modified by K48-linked polyubiquitination remain undetermined. Identifying these critical sites would provide a more precise understanding of the ubiquitination mechanism and potentially reveal new regulatory nodes within this pathway. Secondly, molecular docking analysis predicted the potential interaction interface between PSMD12 and CDK1. As a common approach for exploring protein-protein interactions, however, the predicted results require further experimental validation to elucidate the key domains and interaction sites mediating the physical binding of the two proteins. Future studies could preliminarily screen out the key domains and amino acid residues involved in the interaction. This could be achieved by performing deletion mutations on the predicted binding regions, conducting site-directed mutations on the predicted sites, and integrating immunoprecipitation experiments. Based on these findings, X-ray crystallography or cryo-electron microscopy could be employed to analyze the three-dimensional structure of the complex. Such analyses would intuitively reveal the precise molecular conformation of the interaction, thereby systematically clarifying the molecular basis of PSMD12-CDK1 interaction. Addressing these limitations will be crucial for fully deciphering the regulatory mechanism underlying PSMD12-CDK1 interaction and its implications for HCC progression.

## 5 Conclusion

In conclusion, this study highlights the elevated expression of PSMD12 in HCC, which correlates with poor prognosis, suggesting its potential as a prognostic biomarker for HCC. Mechanistically, PSMD12 regulates CDK1 protein stability through deubiquitination, thereby promoting HCC progression. Therefore, targeting the PSMD12-CDK1 axis may offer a promising therapeutic strategy for HCC.

## Data Availability

Publicly available datasets were analyzed in this study. This data can be found here: The Cancer Genome Atlas Program (TCGA, https://portal.gdc.cancer.gov/), Gene Expression Omnibus (GEO, GSE1212148, GSE14520; https://www.ncbi.nlm.nih.gov/geo/), and the International Cancer Genomics Consortium (ICGC, https://dcc.icgc.org/) databases.

## Supplementary material

Supplementary Figure 1 PSMD12 expression levels and transfection efficiency in HCC cell lines. **(A, B)** The mRNA and protein expression levels of PSMD12 in five HCC cell lines and the immortalized THLE-2 line. **(C, D)** PSMD12 mRNA and protein levels were assessed in MHCC97H cells transfected with shNC or shPSMD12 by qRT-PCR and Western blot with GAPDH as a loading control. **(E, F)** PSMD12 mRNA and protein levels were analyzed in HCCLM3 cells transfected with Vector or PSMD12 by qRT-PCR and Western blot, using GAPDH as a loading control. Data are presented as mean ± SD from triplicate experiments and were analyzed using Student’s t-test, *p < 0.05, **p < 0.01, *** p < 0.001.

Supplementary Figure 2 Gene set enrichment analysis of PSMD12-related genes is enriched in the PI3K/AKT/mTOR signaling pathway. **(A)** Demonstration of enrichment in the PI3K/AKT/mTOR signaling pathway. **(B)** Correlation between the expression of PSMD12 and the PI3K/AKT/mTOR signaling pathway.
