# Supplementary material for: PSMD12 promotes hepatocellular carcinoma progression by stabilizing CDK1
Source: Front Immunol. 2025 Jun 4;16:1581398. doi: 10.3389/fimmu.2025.1581398 (PMC12174133; doi:10.3389/fimmu.2025.1581398)
Supplement: Supplementary file 1 [file DataSheet1.docx]

| **Supplementary Table 1. Abbreviations** | |
| --- | --- |
| **Abbreviation** | **Full name** |
| ACC | Adrenocortical carcinoma |
| ALL | Acute Lymphoblastic Leukemia |
| BLCA | Bladder Urothelial Carcinoma |
| BRCA | Breast invasive carcinoma |
| CDK1 | Cell cycle protein-dependent protein kinase 1 |
| CESC | Cervical squamous cell carcinoma and endocervical adenocarcinoma |
| CHOL | Cholangiocarcinoma |
| CHX | Cycloheximide |
| COAD | Colon adenocarcinoma |
| COADREAD | Colon adenocarcinoma/Rectum adenocarcinoma Esophageal carcinoma |
| Co-IP | Co-immunoprecipitation |
| DFI | Disease-free survival |
| DSS | Disease-specific survival |
| ESCA | Esophageal carcinoma |
| GBM | Glioblastoma multiforme |
| GBMLGG | Glioma |
| GO | Gene Ontology |
| GSEA | Gene set enrichment analysis |
| H&E | Hematoxylin and Eosin staining |
| HCC/LIHC | Hepatocellular carcinoma |
| HNSC | Head and Neck squamous cell carcinoma |
| IF | Immunofluorescence |
| IHC | Immunohistochemistry |
| KEGG | Kyoto Encyclopedia of Genes and Genomes |
| KICH | Kidney Chromophobe |
| KIPAN | Pan-kidney cohort (KICH+KIRC+KIRP) |
| KIRC | Kidney renal clear cell carcinoma |
| KIRP | Kidney renal papillary cell carcinoma |
| LAML | Acute Myeloid Leukemia |
| LC-MS/ MS | liquid chromatogram tandem mass spectrometry |
| LGG | Brain Lower Grade Glioma |
| LUAD | Lung adenocarcinoma |
| LUSC | Lung squamous cell carcinoma |
| OS | Overall survival |
| OV | Ovarian serous cystadenocarcinoma |
| PAAD | Pancreatic adenocarcinoma |
| PCPG | Pheochromocytoma and Paraganglioma |
| PFI | Disease-free interval |
| PRAD | Prostate adenocarcinoma |
| PSMD12 | Proteasome 26S subunit non-ATPase 12 |
| qRT-PCR | Quantitative reverse transcription polymerase chain reaction |
| READ | Rectum adenocarcinoma |
| SKCM | Skin Cutaneous Melanoma |
| STAD | Stomach adenocarcinoma |
| STES | Stomach and Esophageal carcinoma |
| TCGA | The Cancer Genome Atlas |
| TGCT | Testicular Germ Cell Tumors |
| THCA | Thyroid carcinoma |
| THYM | Thymoma |
| UCEC | Uterine Corpus Endometrial Carcinoma |
| UCS | Uterine Carcinosarcoma |
| UPS | Ubiquitin-proteasome system |
| UVM | Uveal Melanoma |
| WB | Western Blot |
| WT | High-Risk Wilms Tumor |
