# Supplementary material for: PSMD12 promotes hepatocellular carcinoma progression by stabilizing CDK1
Source: Front Immunol. 2025 Jun 4;16:1581398. doi: 10.3389/fimmu.2025.1581398 (PMC12174133; doi:10.3389/fimmu.2025.1581398)
Supplement: Supplementary file 2 [file DataSheet2.docx]

| **Primers for real-time PCR** | **Sequences** |
| --- | --- |
| CDK1-Forward primer: | 5'-AAACTACAGGTCAAGTGGTAGCC-3′ |
| CDK1-Reversed primer: | 5'-TCCTGCATAAGCACATCCTGA-3′ |
| PSMD12- Forward primer: | 5'-GACATCCCGTATCTTAGTTGCAG-3′ |
| PSMD12-Reversed primer: | 5'-CCTTCGGTAACCATTCGTAGAGT-3′ |
| GAPDH Forward primer: | 5'-AGAAGGCTGGGGCTCATTTG-3′ |
| GAPDH Reversed primer: | 5'-AGGGGCCATCCACAGTCTTC-3′ |

**Supplementary Table 2. Primers target sequences.**
