# Supplementary figures and images for: PSMD12 promotes hepatocellular carcinoma progression by stabilizing CDK1
Source: Front Immunol. 2025 Jun 4;16:1581398. doi: 10.3389/fimmu.2025.1581398 (PMC12174133; doi:10.3389/fimmu.2025.1581398)

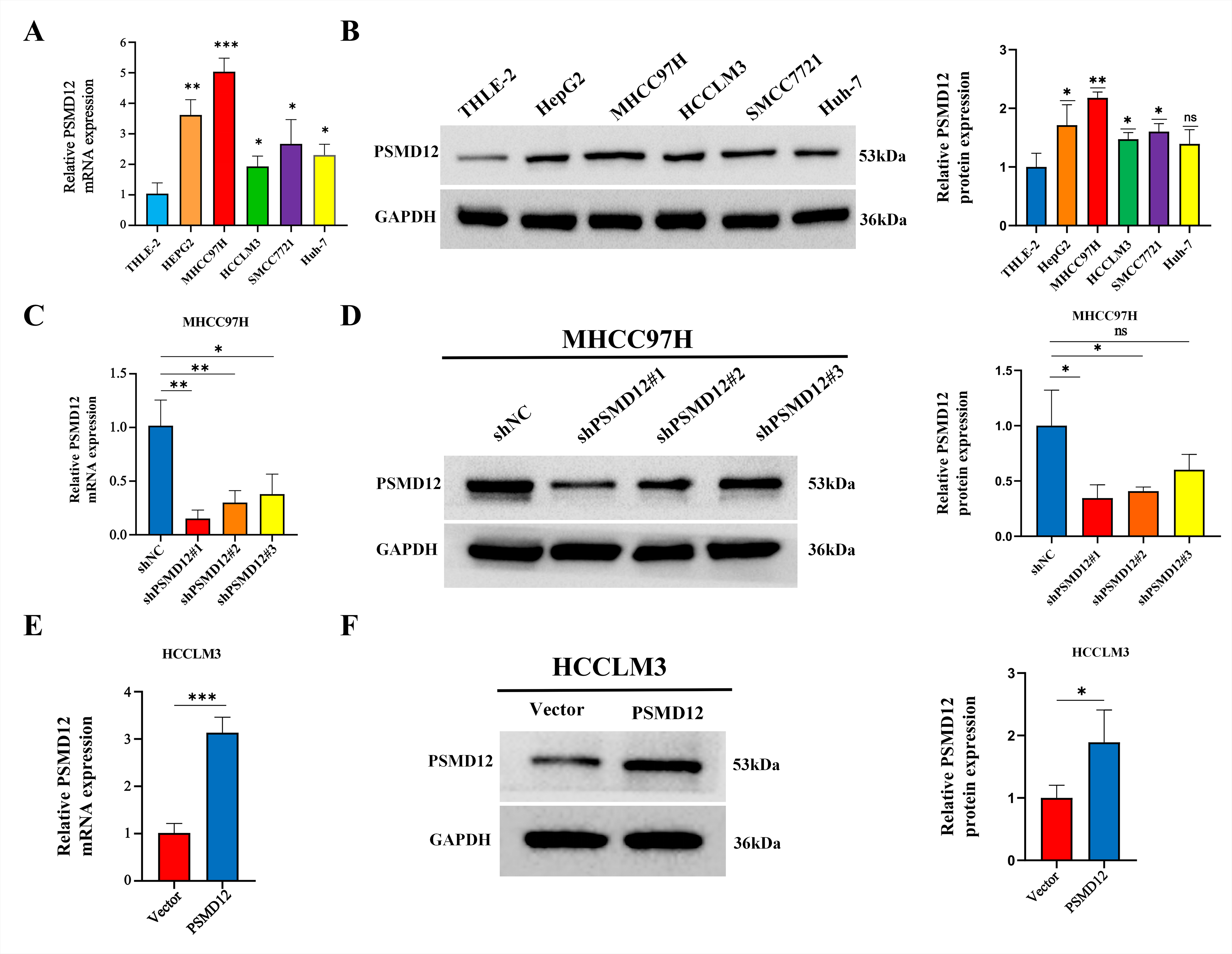

Supplement: Supplementary Figure 1 — PSMD12 expression levels and transfection efficiency in HCC cell lines. (A, B) The mRNA and protein expression levels of PSMD12 in five HCC cell lines and the immortalized THLE-2 line. (C, D) PSMD12 mRNA and protein levels were assessed in MHCC97H cells transfected with shNC or shPSMD12 by qRT-PCR and Western blot with GAPDH as a loading control. (E, F) PSMD12 mRNA and protein levels were analyzed in HCCLM3 cells transfected with Vector or PSMD12 by qRT-PCR and Western blot, using GAPDH as a loading control. Data are presented as mean ± SD from triplicate experiments and were analyzed using Student’s t-test, *p < 0.05, **p < 0.01, *** p < 0.001. [file Image1.tif]

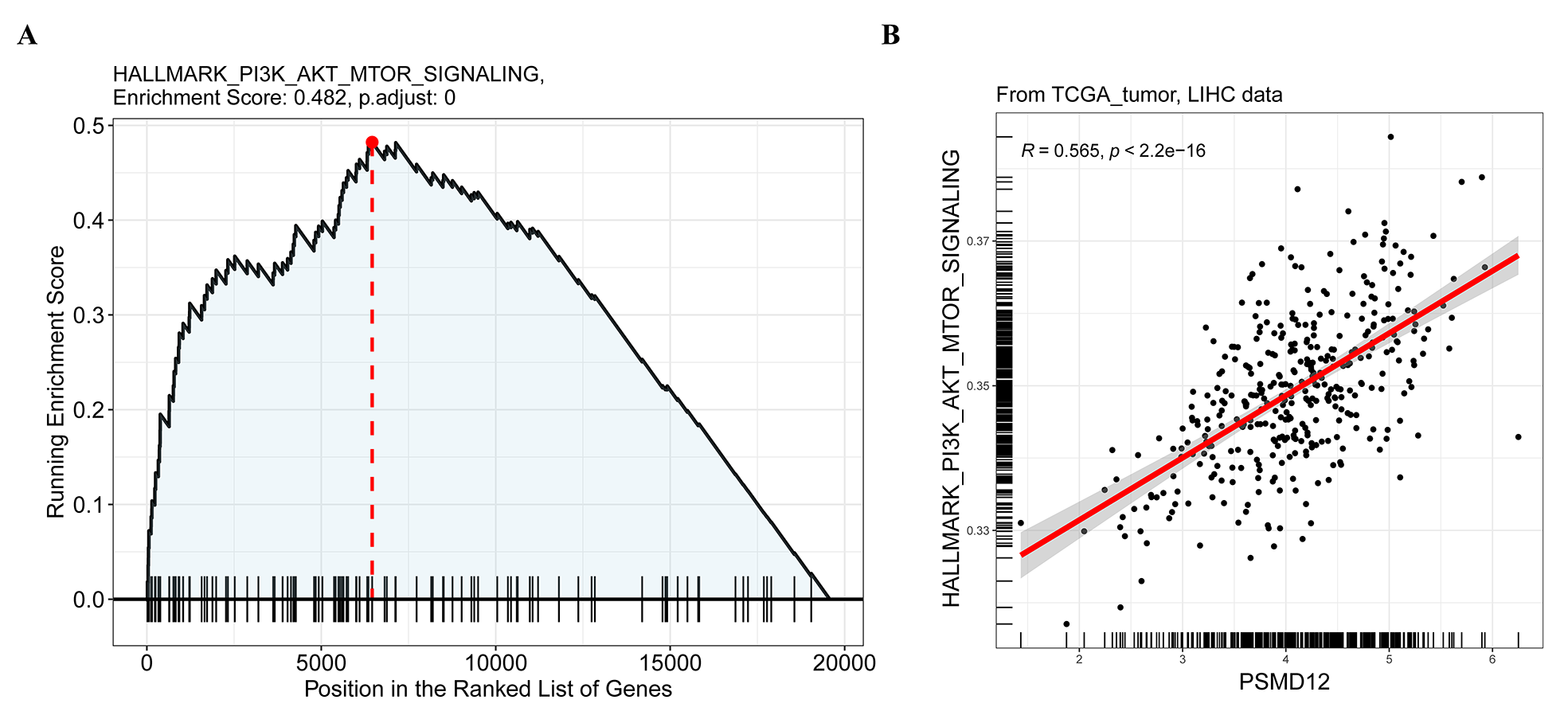

Supplement: Supplementary Figure 2 — Gene set enrichment analysis of PSMD12-related genes is enriched in the PI3K/AKT/mTOR signaling pathway. (A) Demonstration of enrichment in the PI3K/AKT/mTOR signaling pathway. (B) Correlation between the expression of PSMD12 and the PI3K/AKT/mTOR signaling pathway. [file Image2.tif]
